# Supplementary material for: Stimulation of Fibronectin Matrix Assembly by Lysine Acetylation
Source: Cells. 2020 Mar 8;9(3):655. doi: 10.3390/cells9030655 (PMC7140634; doi:10.3390/cells9030655)
Supplement: Supplementary file 1 [file cells-09-00655-s001.pdf]

Supplemental Materials

Cells

Stimulation of fibronectin matrix assembly by lysine acetylation

Maria E. Vega, et al. 2020

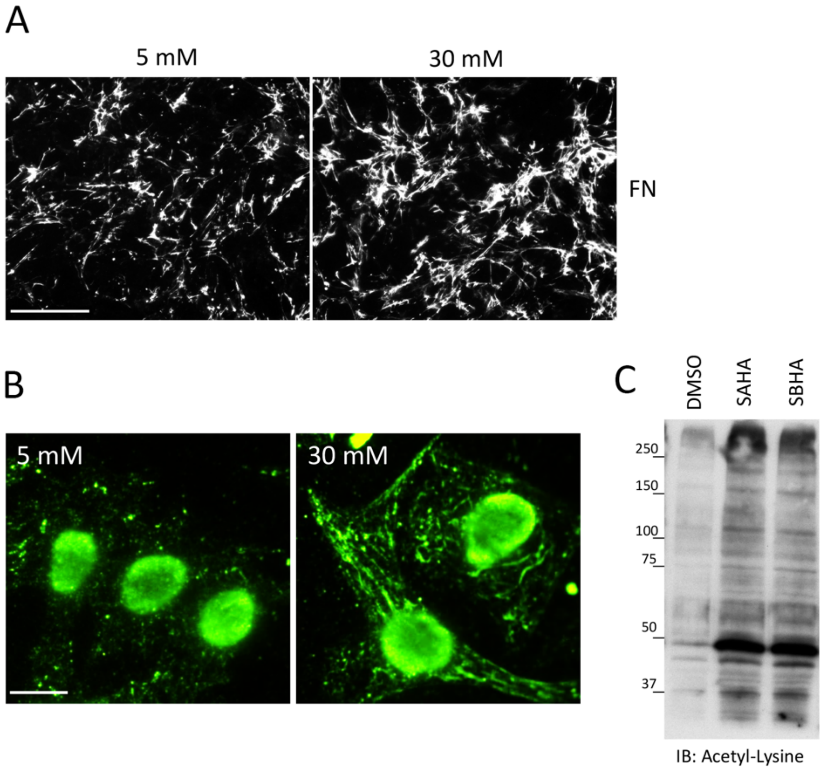

Supplemental Figure 1. Increased FN matrix and acetylation in mesangial cells grown in elevated glucose or treated with deacetylase inhibitor. (A) Mesangial cells were grown in 5 mM or 30 mM glucose with 10 µg/mL human plasma FN for 48 h and stained with anti-FN monoclonal antibody HFN7.1. Scale bar = 50 µm. (B) Mesangial cells grown in 5 mM or 30 mM glucose for 24 h were seeded on 10 µg/mL plasma FN coated glass-bottom dish for 2 h before staining with anti-acetyl-lysine antibody and imaging by TIRF microscopy. Scale bar = 10 µm. (C) Mesangial cells grown in 30 mM glucose were treated with 5 µM SAHA or SBHA or vehicle (DMSO) for 24 h before lysis in DOC buffer. Equal amounts of protein in DOC-soluble fractions were immunoblotted with anti-acetyl-lysine antibodies.

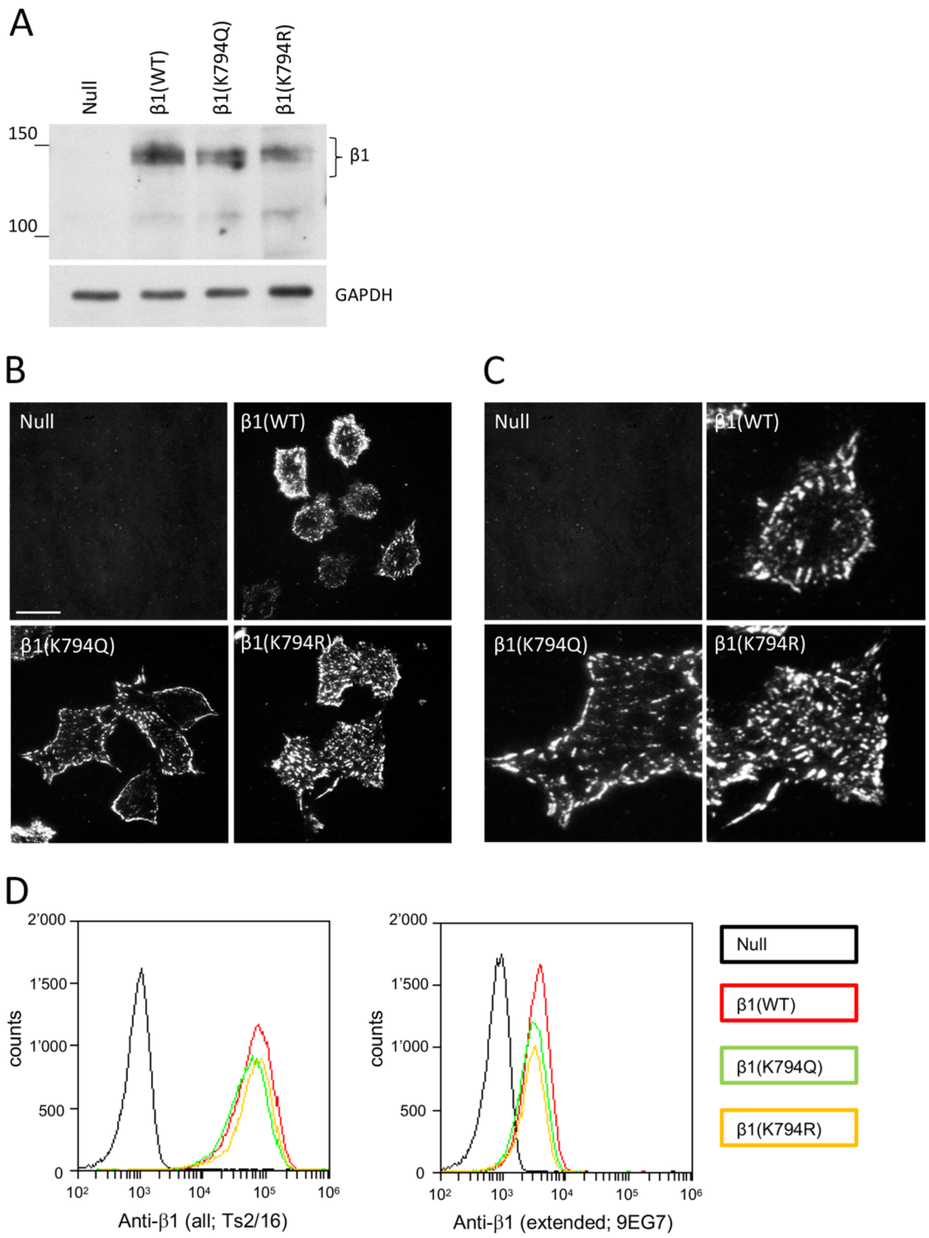

Supplemental Figure 2. Integrin β1 mimetic expression in GD25 integrin β1 null fibroblasts. (A) Whole cell lysates from GD25 β1(null), β1(WT) and mutants β1(K794Q) and β1(K794R) were separated in a 6% polyacrylamide-SDS gel and immunoblotted with antibodies against integrin β1 (top). The GAPDH control immunoblot is shown below. (B) TIRF microscopy of GFP-tagged integrins shows localization to focal adhesions. Scale bar = 10 µm. (C) Enlarged images of individual cells from panel B are shown. (D) Cell surface expression analysis by flow cytometry of β1 integrins in stably transfected GD25 cells labelled with mouse anti-human β1-integrin mAb Ts2/16, recognizing all surface expressed integrins (left panel), or with rat anti-β1-integrin mAb 9EG7 recognizing extended or activated forms (right panel). Curves represent total cell counts, β1(null) in black, β1(WT) in red, β1(K794Q) in green and β1(K794R) in yellow.

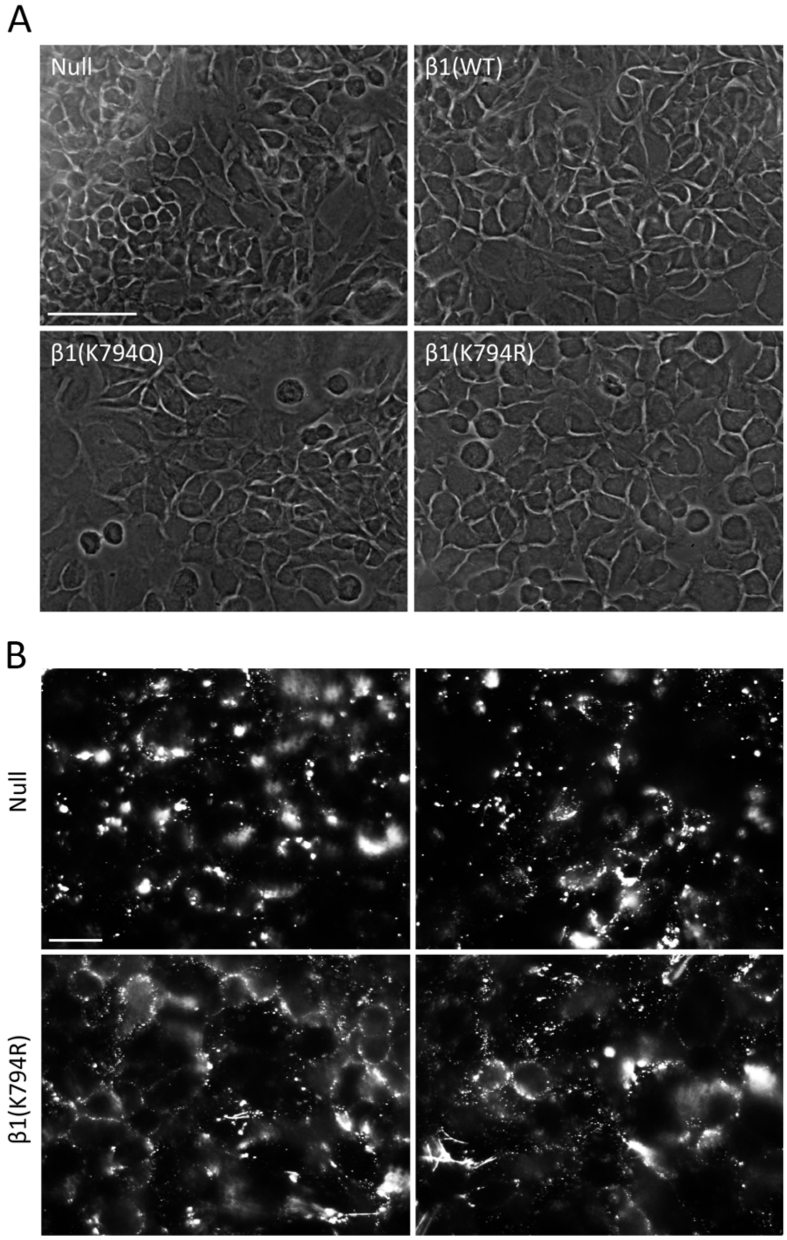

Supplemental Figure 3. Integrin β1(null) and β1(K794R) non-acetylated mimetic cells lack fibrillar FN matrix. (A) Phase contrast images of GD25 cell fields from Figure 2C show that cells are at equivalent densities. Scale bar = 50 μm (B) Higher magnification images of GD25 β1(null) (top) and β1(K794R) (bottom) cells stained with anti-FN antibodies (as in Figure 2C) show FN aggregates and the lack of FN fibrils. Scale bar = 10 μm.

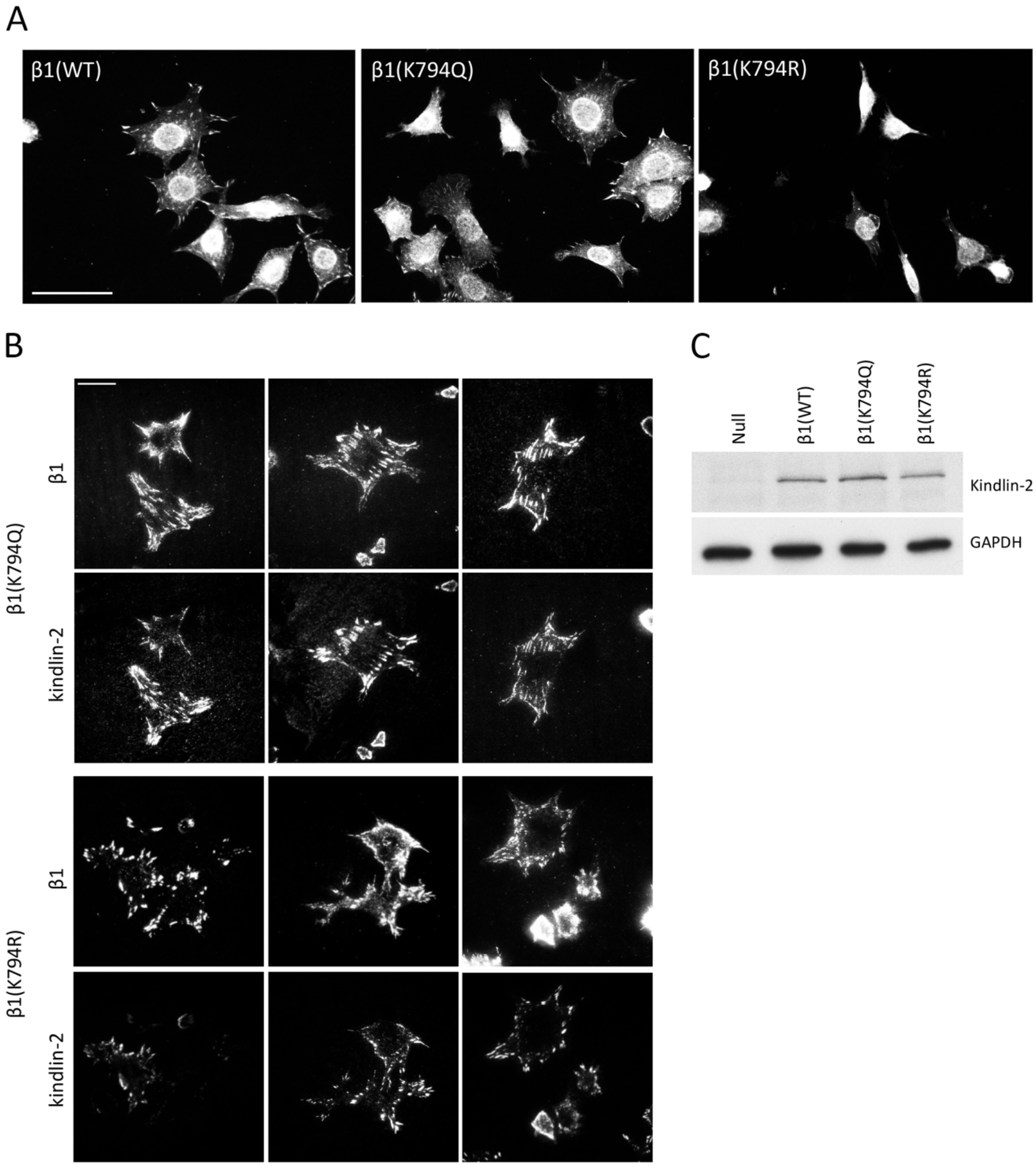

Supplemental Figure 4. Kindlin-2 localization at focal adhesions. (A) GD25 cells seeded on a FN-coated surface were allowed to spread for 4 h and then fixed and stained with anti-kindlin-2 antibodies. Kindlin-2 localization was visualized by epifluorescence microscopy. Scale bar = 50 μm. (B) TIRF microscopy was used to visualize immunostained kindlin-2 and GFP-tagged β1 integrin in β1(K794Q) (top) and β1(K794R) (bottom) cells on a FN-coated surface. Scale bar = 10 μm. (C) Whole cell lysates from GD25 β1(null), β1(WT) and mutants β1(K794Q) and β1(K794R) were immunoblotted with antibodies against kindlin-2 (top) and GAPDH (bottom). The kindlin-2 protein level was lower in GD25 β1(null) cells compared to other GD25 β1 transfected cell lines.
